# The Typhus Situation

**Published:** 1921-05-28

**Authors:** 


### THE PUBLIC WELL-BEING.

### The Typhus Situation.

In this country the amount of publicity given to the danger of typhus through infection from the East of Europe has been rather too little than too great. On the other hand, the United States newspapers, which seem to suffer from a constitutional inability to do things by halves, have been providing the American public with a first-class "scare" on the subject. The extraordinary and alarmist statements which have appeared in the New York papers have drawn a complete reply from Surgeon-General Cumming, of the United States Public Health Service, in which he points out that the menace to America from the introduction of typhus from Europe is no greater to-day than it was six months ago. Even before the Armistice he recognised the potentialities of the disease in spreading to the United States, if adequate precautionary measures were not taken when immigration was resumed. During the past year medical officers of the Public Health Service have been stationed at American Consulates at chief European ports of embarkation to supervise measures to be applied against ships and passengers for the prevention of the spread not only of typhus, but also of plague and cholera. While the measures enforced at the European ports have by no means been perfect, their value is indicated in the fact that several hundred thousand immigrants have come from typhus-infected areas on several hundred ships, and that out of all this number typhus infection occurred only on eight vessels. With one exception infection on the ships was detected by the quarantine officer at New York and effective precautionary measures applied.

The system of quarantine protection developed by the Public Health Service consists of a double line of defence: first, the medical officers at foreign ports who supervise preventive measures specified in the United States Quarantine Regulations, and, second, the facilities at United States quarantine stations. If infection evades the first barrier, the ship still has to undergo inspection and treatment at her American port of arrival. As early as January 17 quarantine officers at Atlantic ports were advised that on account of the unsatisfactory delousing procedure carried out at Danzig all passengers arriving at their ports should be held in quarantine and treated for the destruction of vermin. It must, therefore, be evident to any fair-minded person, the Surgeon-General thinks, that the Federal health authorities have been most diligent in carrying out anti-typhus measures, and that any statement that either the Federal Health Service or the State officials of New York have been derelict or indifferent to the typhus situation is obviously untrue.

As to the statement that "one infected immigrant might spread a plague that would cause a million deaths in six weeks in New York," it is interesting to note that, while not generally known, typhus fever has existed in New York City for years. About 1910 Dr. Nathan Brill recorded a series of somewhat less than 200 cases which he had observed in the previous ten years, and during 1911 thirty-four such cases occurred in New York City, and others have occurred from time to time since that date. For the most part they were of isolated occurrence, and indicated that conditions in New York City were not conducive to any serious spread of the infection. Goldberger and Anderson, of the United States Public Health Service, in 1911, demonstrated by laboratory tests that the so-called "Brill's disease" is identical with old-world typhus; that the clinical manifestations are very similar, but much milder in type; that the disease is transmitted by the louse in the same way as old-world typhus, and while not so virulent, sometimes results fatally.

We cannot afford to ignore the typhus peril. But to make a "scare" of it is merely silly.

